# Supplementary material for: Mechanisms governing the pioneering and redistribution capabilities of the non-classical pioneer PU.1
Source: Nat Commun. 2020 Jan 21;11:402. doi: 10.1038/s41467-019-13960-2 (PMC6972792; doi:10.1038/s41467-019-13960-2)
Supplement: Supplementary file 7 — Source data [file 41467_2019_13960_MOESM7_ESM.zip › Source_Data/FigureS3/FigureS3H_MotifScanOutput/homerResults/motif42.similar.html]

motif42

## Information for motif42

C
A
T
G
T
C
A
G
T
C
A
G
C
T
A
G
A
C
T
G
A
T
G
C
A
G
T
C
A
G
T
C
A
G
T
C
  
Reverse Opposite:  

C
T
A
G
C
T
A
G
C
T
A
G
A
T
C
G
A
G
T
C
A
G
T
C
A
G
T
C
A
G
T
C
G
T
A
C
  

|  |  |
| --- | --- |
| p-value: | 1e-22 |
| log p-value: | -5.233e+01 |
| Information Content per bp: | 1.671 |
| Number of Target Sequences with motif | 508.0 |
| Percentage of Target Sequences with motif | 13.14% |
| Number of Background Sequences with motif | 3665.5 |
| Percentage of Background Sequences with motif | 8.18% |
| Average Position of motif in Targets | 384.7 +/- 367.4bp |
| Average Position of motif in Background | 210.0 +/- 133.5bp |
| Strand Bias (log2 ratio + to - strand density) | -0.2 |
| Multiplicity (# of sites on avg that occur together) | 1.24 |
| Motif File: | file (matrix) reverse opposite |

### Similar de novo motifs found

|  |  |  |  |  |  |  |  |
| --- | --- | --- | --- | --- | --- | --- | --- |
| Rank | Match Score | Redundant Motif | P-value | log P-value | % of Targets | % of Background | Motif file |
| 1 | 0.930 | T A C G A C T G C T A G C T A G C T A G A T C G T A G C A G T C G A T C G A T C | 1e-21 | -50.278299 | 13.53% | 8.59% | motif file (matrix) |
| 2 | 0.888 | C T A G A C T G A T C G T A G C G A T C T A G C A G T C C A T G | 1e-17 | -40.782667 | 14.25% | 9.65% | motif file (matrix) |
| 3 | 0.922 | C T G A A C T G A C T G A G T C A G T C A G T C A T G C | 1e-15 | -36.166169 | 18.55% | 13.62% | motif file (matrix) |
